# Effects of Strength and Distribution of SiC on the Mechanical Properties of SiCp/Al Composites

**DOI:** 10.3390/ma15041288

**Published:** 2022-02-09

**Authors:** Yanju Wang, Wei Wei, Xiaolei He, Xiang Lan, Aixue Sha, Wenfeng Hao

**Affiliations:** 1 Materials Evaluation Center for Aeronautical and Aeroengine Application, AECC Beijing Institute of Aeronautical Materials, Beijing 100095, China; yjwangbiam@163.com (Y.W.); zhaopengtao1980@sina.com (X.L.); aixueshaacaa@sohu.com (A.S.); 2 Faculty of Civil Engineering and Mechanics, Jiangsu University, Zhenjiang 212013, China; wgwuvip@163.com; 3 Institute of Aluminum Alloys, AECC Beijing Institute of Aeronautical Materials, Beijing 100095, China; liuyh17vip@163.com; 4 College of Mechanical Engineering, Yangzhou University, Yangzhou 225127, China

**Keywords:** SiCp/Al composites, mechanical properties, strength of particle, distribution of particle, volume fraction of particle, damage model

## Abstract

In this paper, considering the strength and geometric discrete distribution characteristics of composite reinforcement, by introducing the discrete distribution function of reinforcement, the secondary development of ABAQUS is realized by using the Python language, the parametric automatic generation method of representative volume elements of particle-reinforced composites is established, and the tensile properties of silicon carbide particle-reinforced aluminum matrix composites are analyzed. The effects of particle strength, particle volume fraction, and particle random distribution on the mechanical properties of SiCp/Al composites are studied. The results show that the random distribution of particles and the change in particle strength have no obvious influence on the stress–strain relationship before the beginning of material damage, but have a great influence on the damage stage, maximum strength, and corresponding failure strain. With the increase in particle volume fraction, the damage intensity of the model increases, and the random distribution of particles has a great influence on the model with a large particle volume fraction. The results can provide a reference for the design, preparation, and characterization of particle-reinforced metal matrix composites.

## 1. Introduction

Silicon carbide particle-reinforced aluminum matrix (SiCp/Al) composites are favored in the fields of microelectronic packaging, aircraft, high-speed trains, armor protection, and so on because of their excellent properties, such as high specific strength, high modulus, excellent thermal conductivity, wear resistance, and low coefficient of thermal expansion [1,2,3,4]. Therefore, it is of great engineering value to study the mechanical properties of SiCp/Al composites. The greatest characteristic of SiCp/Al composites is the designability of their functions and properties. The mechanical and thermophysical properties of the composites can be changed by selecting a different matrix, reinforcement, volume fraction of reinforcement particles, particle shape, and particle size. One of the most common methods to study the strengthening mechanism and theoretical prediction of SiCp/Al composites is finite element numerical simulation. The finite element method can obtain the complete stress and strain field at the meso scale to reflect the macro-response characteristics of the composites, so as to quantitatively analyze the dependence of the macro-properties of the composites on the mesostructure [5,6,7].

Metal matrix composites are becoming increasingly widely used in engineering fields. If the relationship between deformation and damage evolution of metal matrix composites and mesostructures are obtained, it is possible to optimize the design of advanced metal matrix composites according to external loads [8,9,10]. Because of the high cost of experimental research, finite element numerical simulation is widely used to study the relationship between the macro-mechanical properties and the microstructure of metal matrix composites. Shao et al. [11] established a finite element numerical model considering the particle size parameters of reinforcement, and studied the effect of particle size on the mechanical behavior of composites. Qing et al. [12,13,14] studied the effects of particle arrangement and interface strength on the mechanical properties of particle-reinforced metal matrix composites under different loading modes (uniaxial and biaxial tension), and developed a program to automatically generate a two-dimensional model with random distribution of particle size and position. In the usdfld subroutine of ABAQUS, the elastic damage model based on the maximum principal stress criterion and the plastic damage model based on the stress triaxial index are developed, respectively. Yan et al. [15] used the two-dimensional multi-particle random distribution cell model to simulate the mechanical behavior of composites in a thermal residual stress field and superimposed residual stress field, and proposed a method to determine the yield strength of composites. Yan et al. [15] studied the effect of particle size on the deformation behavior of metal matrix composites by using the finite element method, and clearly evaluated the contribution of various strengthening mechanisms to the overall composite strengthening and the effect of particle size on various mechanisms. Zhang et al. [16,17] studied the effect of a ductile interface on the strengthening behavior of particle-reinforced metal matrix composites through numerical simulation, in which the stiffness, thickness, and debonding position of the interface were considered. Geni and Kikuchi [18] numerically simulated the fracture process of SiC particle-reinforced aluminum alloy by the damage mechanics method, and studied the effects of the uneven distribution of the SiC particle volume fraction and aspect ratio on the matrix. Zhou et al. derived a non-thick interface layer element by using the cohesion model, and directly reflected the properties of the interface layer in the relationship between the bonding force of the interface layer and the relative displacement of the upper and lower surfaces of the interface layer. Chawla et al. [19] studied the three-dimensional (3D) microstructure and the mechanical behavior of SiC particle-reinforced aluminum composites using finite element modeling. Sozhamannan et al. [20,21] used two-dimensional finite element analysis to study and predict the strength of particle-reinforced metal matrix composites from the model based on SEM images, and used the model to study the main failure forms, such as matrix yield, interface debonding, and particle fracture. Although the finite element method is widely used in the relevant literature, it is found that the effect of particle strength on the damage evolution of metal matrix composites is not considered.

Therefore, in this paper, considering the strength and geometric discrete distribution characteristics of composite reinforcement, by introducing the discrete distribution function of reinforcement, the secondary development of ABAQUS is realized by using the Python language, the parametric automatic generation method of representative volume elements of particle-reinforced composites is established, and the tensile properties of silicon carbide particle-reinforced aluminum matrix composites are analyzed. The effects of particle strength, particle volume fraction, and particle random distribution on the mechanical properties of SiCp/Al composites are studied.

## 2. Computational Micromechanics Model

In order to study the influence of the mesostructure on the deformation and damage evolution of metal matrix composites, the mesostructure should be able to reflect the randomness of the meso-characteristics and can be changed according to needs. At present, the random sequential adsorption (RSA) algorithm is widely used to generate the microstructure of composites. This method is also used to generate the meso-model of SiC particle-reinforced aluminum matrix composites.

**Figure 1:**
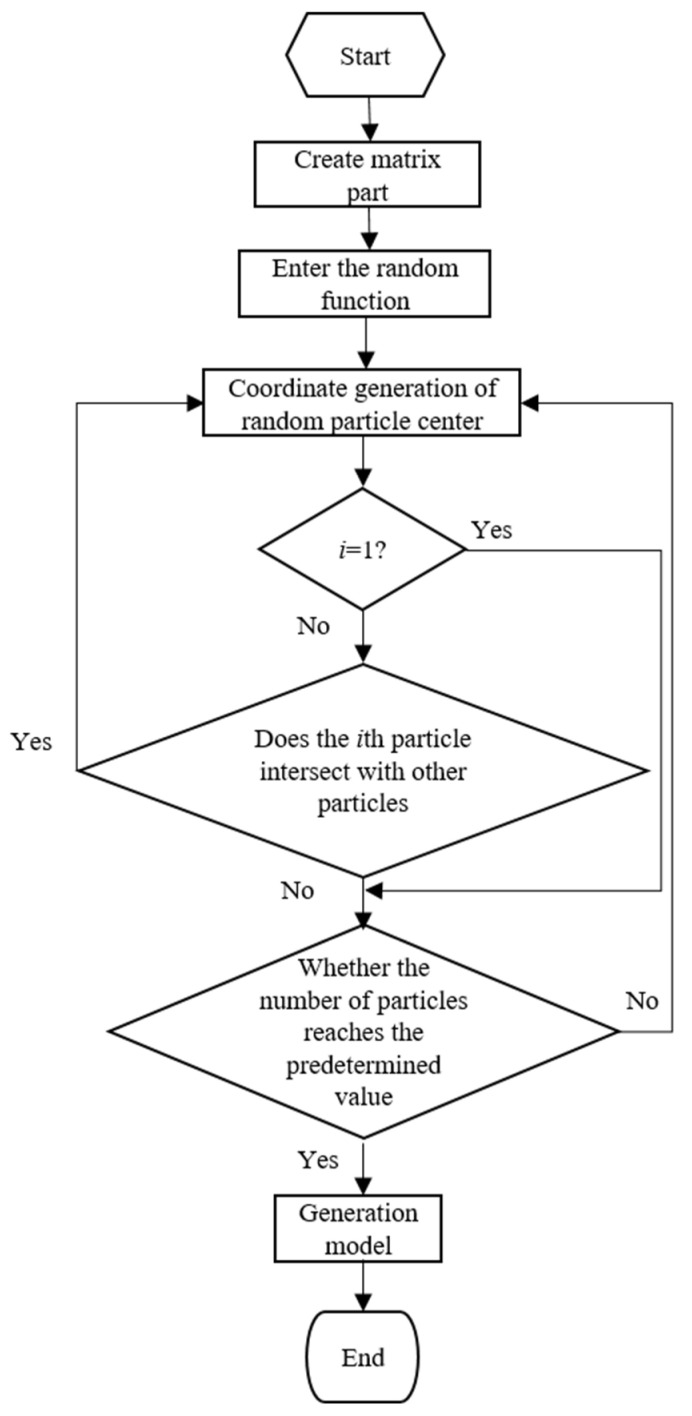
Geometric model modeling process based on RSA method.

Figure 1 shows the process of geometric model modeling of a SiCp/Al composite with random particle distribution based on the RSA method. First, we write a code to generate a two-dimensional matrix part with a square shape, draw a sketch on the matrix, and then import the random function, and use the while loop. In the loop, we first use the random function to generate particle center coordinates at random positions in the square area of the matrix part, and then divide the particle circle area based on the center coordinates. After the number of particles reaches the predetermined value of the while cycle, the cycle ends, the program ends, and ABAQUS generates the CAE model.

In order to avoid the possible intersection of particles in the generated model if the number of particles is large, the distance between each newly generated particle and the existing particle must be greater than a specified value. At the same time, in order to control the quality of mesh generation, the boundary of the new particle distance model must also meet certain conditions. If the above conditions are not met, the position coordinates of the particle will be randomly generated by the random function again until all the conditions are met. The basic idea of the particle intersection judgment algorithm is as follows:

1. Create two lists, List1 = {} and List2 = {}, to store the abscissa and ordinate of the center of each particle circle, respectively.
2. The center = (List1 [*i*], List2 [*i*]) of the *i*th particle circle, and the values of List1 [*i*] and List2 [*i*] are randomly generated by the random function.
3. After generating the *i*th particle circle, compare the distance between the center of the particle circle and the center of all (*i* − 1) particle circles generated before. If all distances are greater than the sum of the two circle radii, ABAQUS continues to generate the (*i* + 1) particle. Otherwise, ABAQUS will delete and regenerate the *i*th particle.

**Figure 2:**
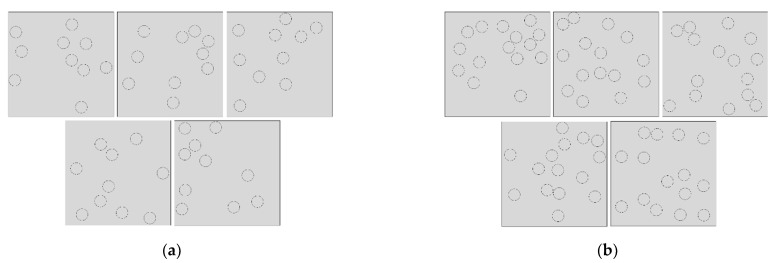
Random distribution model of particles: (**a**) 10-particle models, (**b**) 15-particle models.

In this study, we first generated a meso-model containing 10 equal-sized particles with a volume fraction of 10% and a matrix size of 200 μm × 200 μm. The radius of the particles was *r* = 11.28 μm. Then, keeping the matrix size and particle radius unchanged, we modified the Python script to generate a meso-model containing 15 equal-sized particles with a volume fraction of 15%. In order to eliminate the randomness in the model, we generated five groups of models with uniform random distribution of particles corresponding to two volume fractions, as shown in Figure 2. We applied a uniform tensile load along the *y*-axis. In the model, the interface between the particles and the matrix is not considered, and it is assumed that the particles and the matrix are perfectly bonded together. The global mesh size of the model is 0.01 and the local mesh size on the particle circle is 0.004. The advanced free mesh algorithm based on quadrilateral was adopted for mesh element division, and the element type was plane strain.

In this study, the elastic modulus, Poisson’s ratio, and yield strength of the matrix were 105 GPa, 0.34, and 270 MPa, respectively. The SiC particles were isotropic elastic brittle materials, and the elastic modulus and Poisson’s ratio were 400 GPa and 0.17, respectively. The damage initiation of SiC particles is described by the maximum nominal stress criterion. We developed an ABAQUS/standard subroutine, USRFLD, to realize the failure mechanism of the SiC particles. For brittle failure, the linear damage evolution law is used to describe the behavior after failure. Damage initiation strain *ε*_0_ and damage end strain *ε*_T_ have the following relationship:

$$A=\frac{\varepsilon_{t}-\varepsilon_{0}}{\varepsilon_{0}}$$

**Figure 3:**
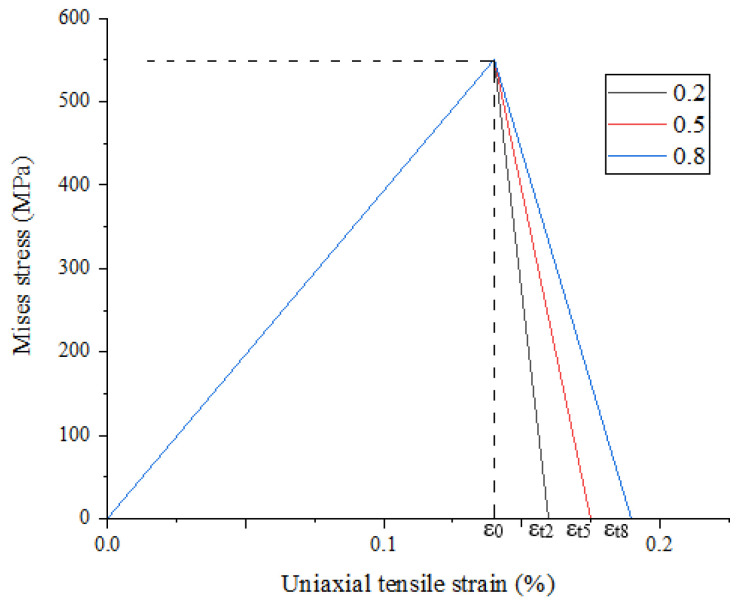
Damage evolution of particles.

If the strain of the SiC particle element is greater than the damage end strain, the Young’s modulus of the element is reduced to 0.1% of the initial value. Figure 3 shows the damage evolution of SiC particles under a tensile load under different critical damage parameters a. Here, we assume A = 0.2.

## 3. Results and Discussion

**Figure 4:**
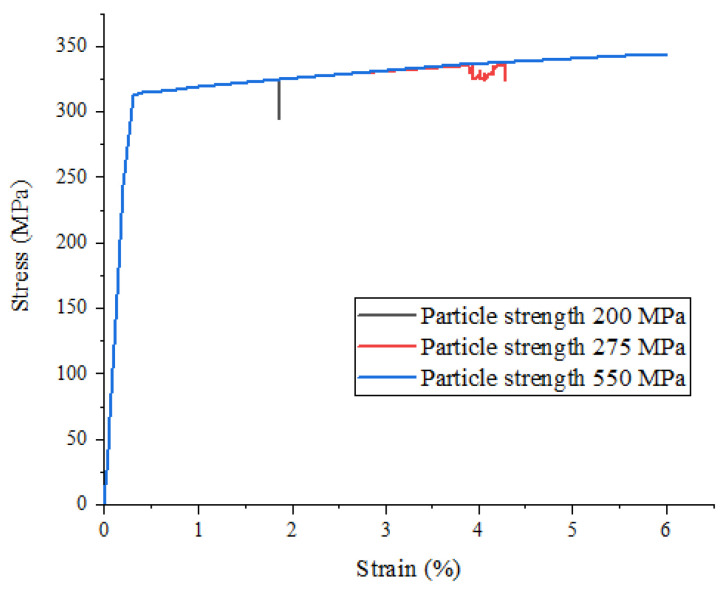
Stress–strain curves of SiCp/Al composites with different particle strengths.

In order to study the effect of particle strength on the mechanical properties of particle-reinforced metal matrix composites under a uniaxial tensile load, we analyzed the stress–strain curves obtained by changing the particle strength to 200 MPa, 275 MPa, and 550 MPa when the volume fraction of the above finite element model was 10%, as shown in Figure 4.

It can be seen from Figure 4 that the change in particle strength has no obvious effect on the stress–strain relationship before the beginning of material damage, but when the material enters the damage and failure stage, the maximum strength and failure strain of the material gradually increase with the continuous increase in particle strength.

In order to eliminate the randomness in the model, we simulated five groups of models with uniform random distribution of particles corresponding to different particle strengths, and obtained the stress–strain curve under uniaxial tension.

**Figure 5:**
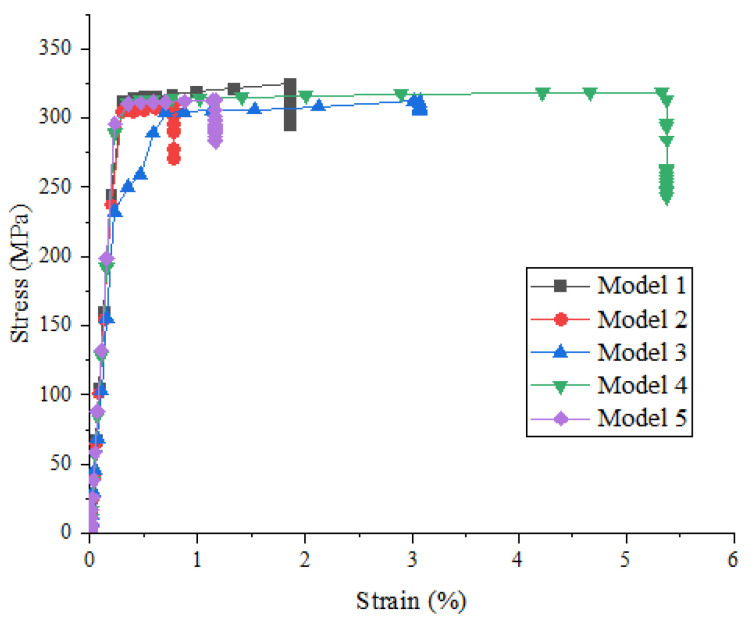
Stress–strain curves of SiCp/Al composites with different models (particle volume fraction is 10% and particle strength is 200 MPa).

Figure 5 shows the effect of random distribution of particles on the strength and damage evolution process of metal matrix composites when the particle volume fraction is 10% and the particle strength is 200 MPa. It can be seen that the random distribution of particles has no obvious influence on the stress–strain relationship before the beginning of material damage, but has a great influence on the damage stage, maximum strength, and corresponding failure strain.

**Figure 6:**
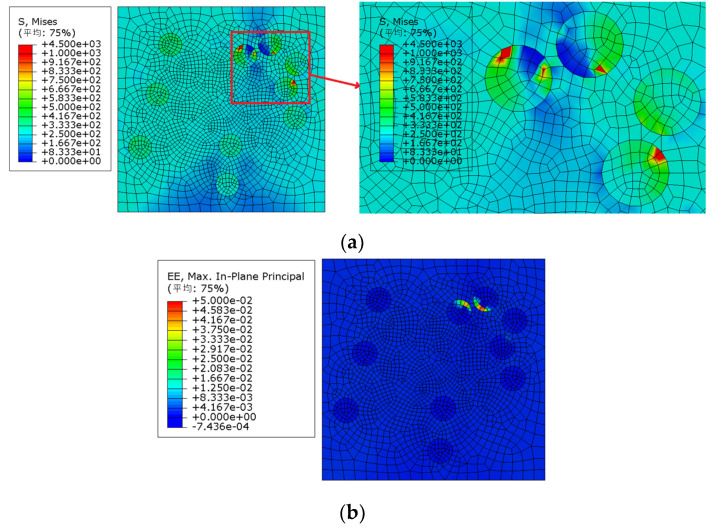
Von Mises stress and strain distribution of model 2 (particle volume fraction is 10% and particle strength is 200 MPa). (**a**) Von Mises stress distribution, (**b**) strain distribution; (平均—Average).

It can be seen from Figure 5 that model 2 was first destroyed, and the failure strain was approximately 0.78%. The Von Mises stress nephogram of each model under 0.78% strain (as shown in Figure 6a) was extracted for comparative analysis: the maximum Mises stress of model 2 appeared in the particle aggregation area inside the model, and the particle aggregation caused the early failure of model 2.

We extracted the elastic strain nephogram of model 2 under 0.78% strain, as shown in Figure 6b. It can also be observed that the maximum elastic strain of model 2 appears in the particle aggregation area inside the model, and the particle aggregation causes the early failure of model 2.

**Figure 7:**
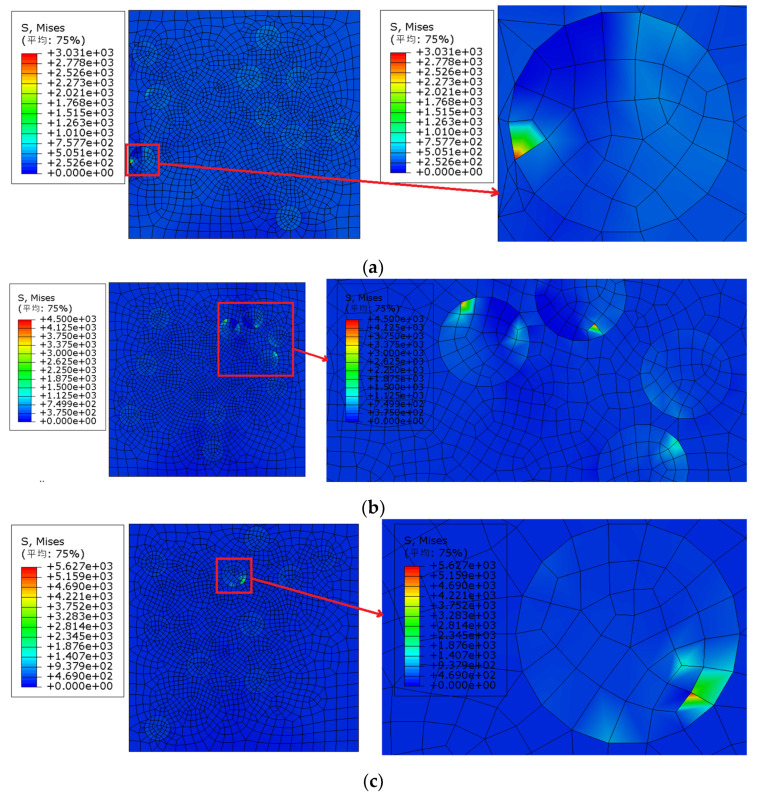

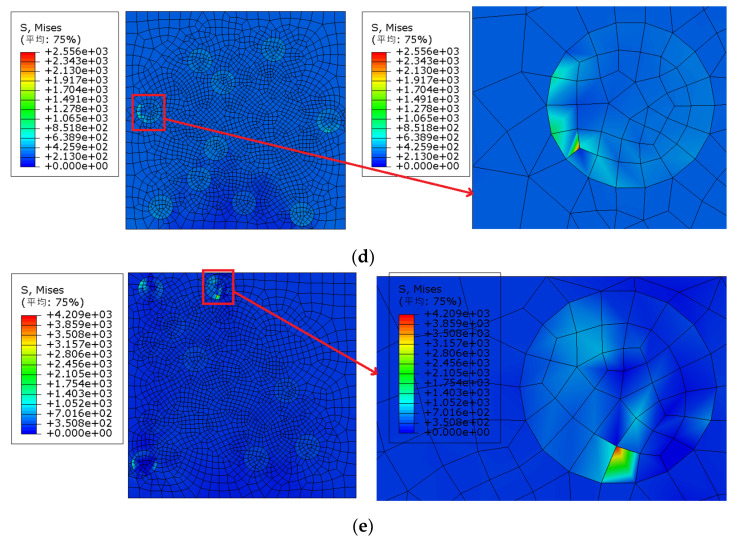
Von Mises stress distribution and local enlarged view under failure strain (particle volume fraction is 10% and particle strength is 200 MPa): (**a**) Model 1 (strain 1.86%), (**b**) Model 2 (strain 0.78%), (**c**) Model 3 (strain 3.01%), (**d**) Model 4 (strain 4.22%), (**e**) Model 5 (strain 1.16%); (平均—Average).

In order to better demonstrate the stress distribution of the model, Figure 7a–e list the Von Mises stress nephogram and local enlarged diagram of models 1–5 under their respective failure strain when the particle volume fraction is 10% and the particle strength is 200 MPa. The failure strain of model 1 was approximately 1.86%. The Von Mises stress nephogram of the model under 1.86% strain was extracted for observation and analysis: the maximum Von Mises stress of model 1 appeared at the particles close to the boundary of the model, and the particle edge was the cause of the failure of model 1. The failure strain of model 2 was approximately 0.78%. The Von Mises stress nephogram of the model under 0.78% strain was extracted for observation and analysis: the maximum Von Mises stress of model 2 appeared in the particle aggregation area inside the model, and particle aggregation was the cause of the failure of model 2. Model 3 failed at the latest, and the failure strain was approximately 3.01%. The Von Mises stress nephogram of the model under 3.01% strain was extracted for observation and analysis: the maximum Von Mises stress of model 3 appeared in the particle area inside the model, the particle distribution inside model 3 was more uniform than that of other models, and model 3 failed later than other models. The failure strain of model 4 was approximately 4.22%. The Von Mises stress nephogram of the model under 4.22% strain was extracted for observation and analysis: the maximum Von Mises stress of model 4 appeared at the particles close to the boundary of the model, and the particle edge was the cause of the failure of model 4. The failure strain of model 5 was approximately 1.16%. The Von Mises stress nephogram of the model under 1.16% strain was extracted for observation and analysis: the maximum Von Mises stress of model 5 appeared at the particles close to the boundary of the model, and the particle edge was the cause of the failure of model 5.

**Figure 8:**
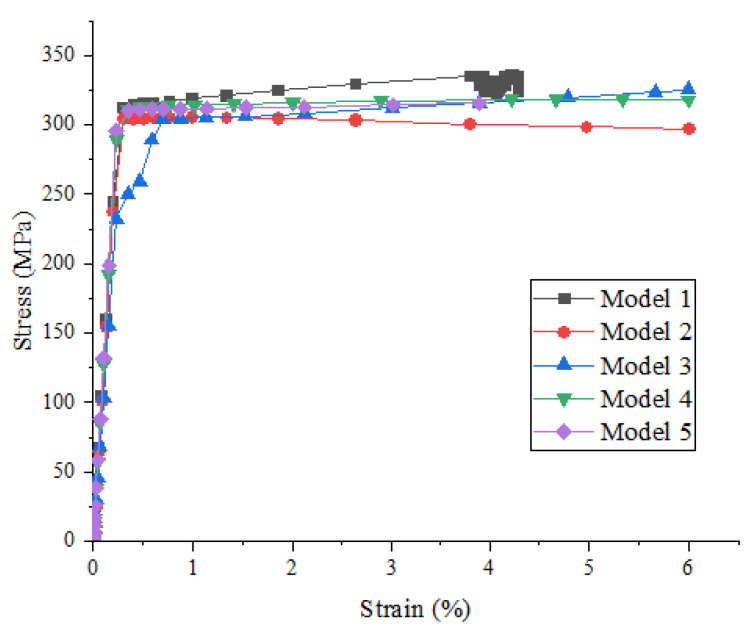
Stress–strain curves of SiCp/Al composites with different models (particle volume fraction is 10% and particle strength is 275 MPa).

Figure 8 shows the effect of the random distribution of particles on the strength and damage evolution process of metal matrix composites when the particle volume fraction is 10% and the particle strength is 275 MPa. It can be seen that the influence of particle random distribution on the stress–strain relationship before the beginning of material damage is not obvious, but the influence on material damage and failure is gradually increasing, i.e., with the continuous increase in strain, the influence of the particle random distribution on the mechanical properties of the materials is increasing, which is shown in the figure, where the curve is increasingly dispersed.

**Figure 9:**
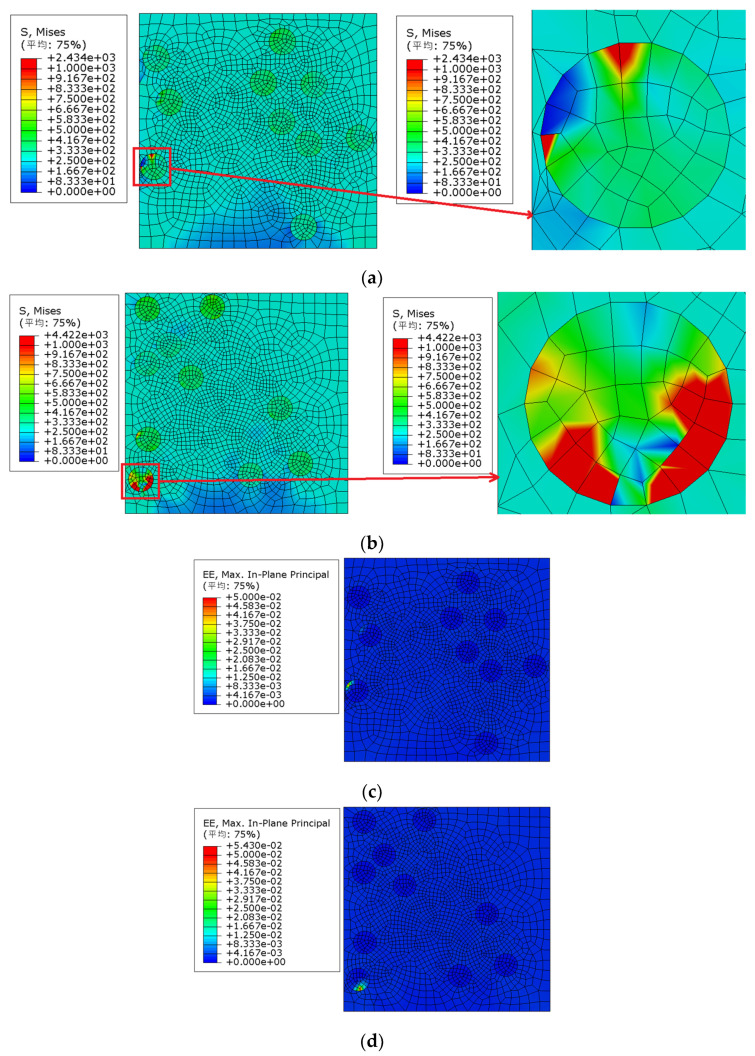
Von Mises stress of SiCp/Al composites with different models (particle volume fraction is 10% and particle strength is 275 MPa): (**a**) Model 1, (**b**) Model 5, (**c**) strain distribution (Model 1), (**d**) strain distribution (Model 5); (平均—Average).

It can be seen from Figure 8 that model 1 and model 5 were damaged in advance, and the failure strain was approximately 3.90%. The Von Mises stress nephogram of each model under 3.90% strain (as shown in Figure 9a,b) was extracted for comparative analysis: the maximum Von Mises stress of model 1 and model 5 appeared at the particles close to the boundary of the model, and the edge of particles caused the early failure of the model.

We extracted the elastic strain nephogram of each model under 3.90% strain, as shown in Figure 9c,d. It can also be observed that the maximum elastic strain of model 1 and model 5 appeared at the particles close to the boundary of the model, and the particle edge caused the early failure of the model.

**Figure 10:**
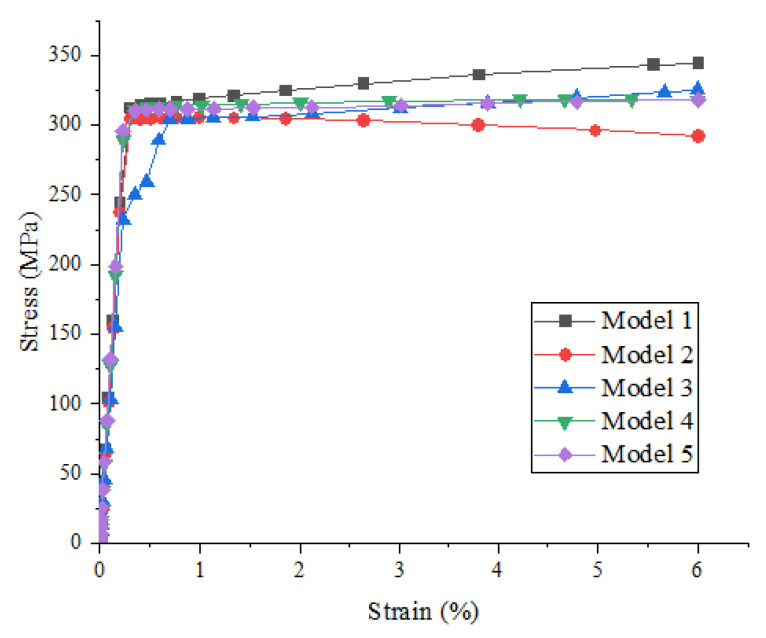
Stress–strain curves of SiCp/Al composites with different models (particle volume fraction is 10% and particle strength is 550 MPa).

Figure 10 shows the effect of the random distribution of particles on the strength and damage evolution process of metal matrix composites when the particle volume fraction is 10% and the particle strength is 550 MPa. It can be seen that the influence of the particle random distribution on the stress–strain relationship before the beginning of material damage was not obvious, but the influence on material damage and failure was gradually increasing, i.e., with the continuous increase in strain, the influence of the particle random distribution on the mechanical properties of materials increased, which is shown in the figure, were the curve is more and more dispersed. When the value of the fixed *x*-axis is 6%, the stresses of model 1-5 in Figure 10 are 344.621 MPa, 291.881 MPa, 325.408 MPa, 318.054 MPa, and 318.362 MPa, respectively.

In order to view the stress distribution of the model conveniently, the stress nephogram of the model needs to be analyzed. The stress nephogram after 6% strain was applied to the model as follows.

**Figure 11:**
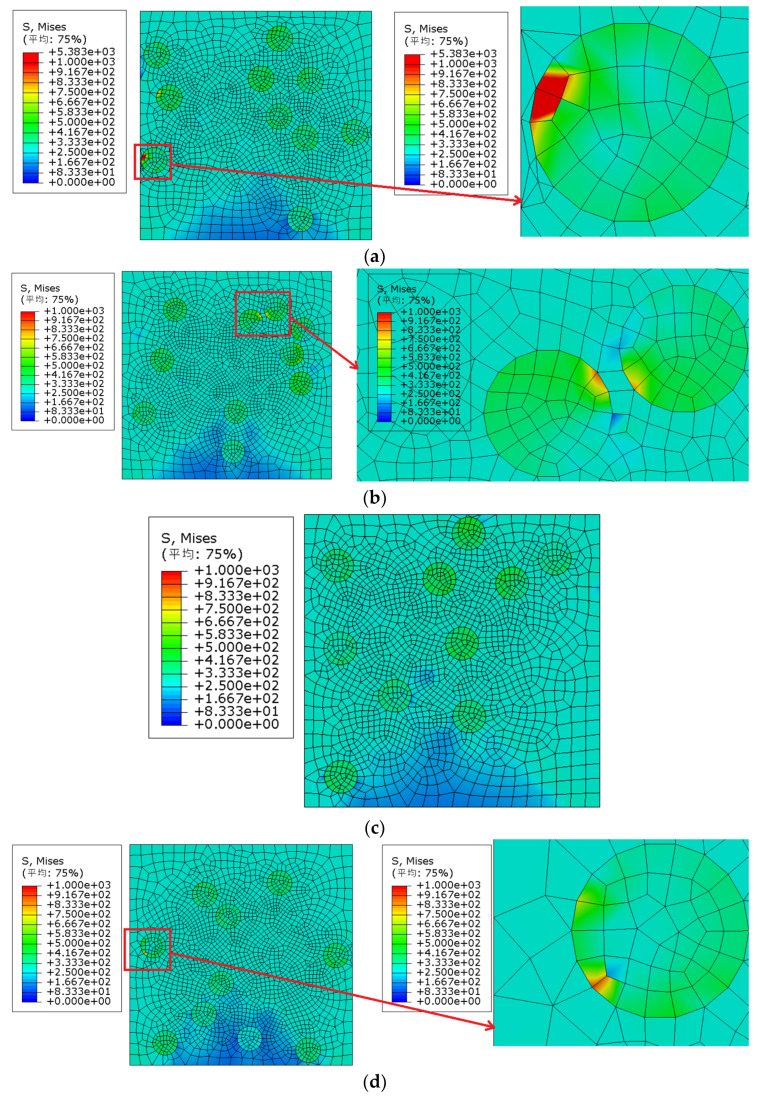

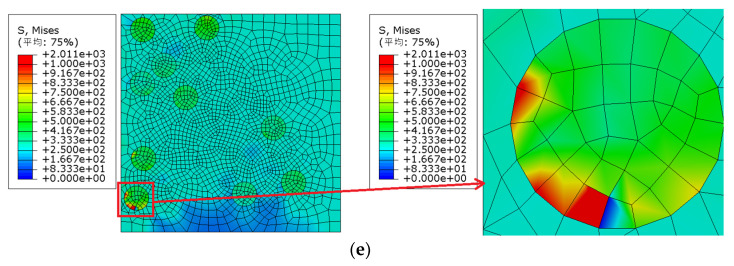
Von Mises stress of SiCp/Al composites with different models (particle volume fraction is 10% and particle strength is 550 MPa): (**a**) Model 1, (**b**) Model 2, (**c**) Model 3, (**d**) Model 4, (**e**) Model 5; (平均—Average).

Figure 11a–e, respectively, show the stress nephograms of five groups of different particle distribution models. It can be seen from the figure that the maximum Von Mises stress of models 1, 4, and 5 appears at the particles close to the boundary of the model, and the maximum Von Mises stress of model 2 appears in the particle aggregation area inside the model. Both particle aggregation and particle edge will affect the damage evolution of the model.

In order to better explain the process of particle damage evolution, the effects of particle volume fraction on the strength and damage evolution of metal matrix composites were further studied in this research. Two cases with a particle volume fraction of 10% and 15% were analyzed. Since the particle volume fraction of 10% has been analyzed above, the particle volume fraction was increased to 15% by modifying the Python script, i.e., the number of particles was increased to 15, and the radius of particles was kept constant at *r* = 11.28 μm. Five groups of models with uniform random distribution of particles were simulated to eliminate the randomness, and the stress–strain curve under uniaxial tension was obtained.

**Figure 12:**
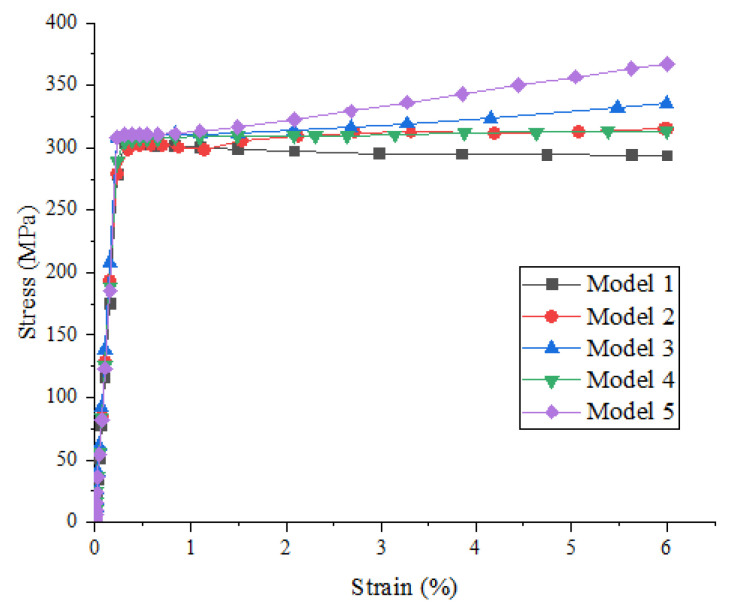
Stress–strain curves of SiCp/Al composites with different models (particle volume fraction is 15% and particle strength is 550 MPa).

Figure 12 shows the effect of the random distribution of particles on the strength and damage evolution process of metal matrix composites when the particle volume fraction is 15% and the particle strength is 550 MPa. Comparing Figure 12 with Figure 10, the effect of the particle random distribution on the stress–strain relationship before material damage is not obvious, but the effect on material damage and failure is gradually increasing, i.e., with the continuous increase in strain, the effect of the particle random distribution on the mechanical properties of materials increased, which is shown in the figure, where the curve is more and more dispersed. The model with a large particle volume fraction has a great influence on the random distribution of particles. When the value of the fixed *x*-axis is 6%, the stresses of model 1–5 in Figure 12 are 293.531 MPa, 315.691 MPa, 335.468 MPa, 312.846 MPa, and 367.065 MPa, respectively.

Table 1 summarizes the stress and its standard deviation under different particle distribution models when the particle volume fraction is 10% and 15%, the particle strength is 550 MPa, and the strain is 6%. It can be seen from Table 1 that the mean stress of the 10% particle volume fraction is 319.665 MPa and that of the 15% particle volume fraction is 324.920 MPa, indicating that the strength of model damage increases with the increase in the particle volume fraction. However, the standard deviation of stress is very large, indicating that the random distribution of particles has a great influence on the damage strength of the model. With the increase in particle volume fraction, the standard deviation also increases, which indicates that the composites with a higher particle volume fraction are more sensitive to the macro-response strength.

## 4. Conclusions

This paper proposes an algorithm and writes a script to implement the algorithm in Python. After the script is opened and run through the script interface of ABAQUS, the modeling of multiple particles randomly placed in the matrix and disjoint between particles is successfully realized. Based on the two-dimensional finite element analysis of different mesostructures and a series of numerical simulations, the following conclusions are drawn:

1. The change in particle strength has no obvious effect on the stress–strain relationship before the beginning of material damage. After entering the damage stage, the maximum strength and failure strain of the material increase gradually with the increase in particle strength.
2. The effect of particle random distribution on the stress–strain relationship before the beginning of material damage is not obvious, but it has a great influence on the damage stage, maximum strength, and corresponding failure strain.
3. With the increase in particle volume fraction, the damage strength of the model increases. The model with a large particle volume fraction has a great influence on the random distribution of particles.

## Figures and Tables

**Table 1:** Von Mises stresses of SiCp/Al composites with different particle distributions and particle volume fractions (MPa).

Particle Volume Fraction	Model 1	Model 2	Model 3	Model 4	Model 5	Average Value	Standard Deviation
10%	344.62	291.88	325.41	318.05	318.36	319.67	16.92
15%	293.53	315.69	335.47	312.85	367.07	324.92	24.91

## Data Availability

No new data were created or analyzed in this study. Data sharing is not applicable to this article.

## References

[1] Song M., Huang B. (2008). Effects of particle size on the fracture toughness of SiC_p_/Al alloy metal matrix composites. Mater. Sci. Eng. A.

[2] Ekici R., Apalak M.K., Yildirim M., Nair F. (2010). Effects of random particle dispersion and size on the indentation behavior of SiC particle reinforced metal matrix composites. Mater. Des..

[3] Chen X.W., Zhang T.J., Liu S.H. (2009). Accelerated Ageing Behavior of PMMA under Hygrothermal Air and Water Conditions. Fail. Anal. Prev..

[4] Mao D., Meng X., Xie Y., Yang Y., Xu Y., Qin Z., Chang Y., Wan L., Huang Y. (2022). Strength-ductility balance strategy in SiC reinforced aluminum matrix composites via deformation-driven metallurgy. J. Alloys Compd..

[5] Maleki K., Alizadeh A., Hajizamani M. (2020). Compressive strength and wear properties of SiC/Al6061 composites reinforced with high contents of SiC fabricated by pressure-assisted infiltration. Ceram. Int..

[6] Chen X.W., Pei G.L., Jin Y.S. (2009). Study on Accelerated Ageing of Aeronautical Perspex (PMMA) in Ultraviolet. J. Aeronaut. Mater..

[7] Bouafia F., Serier B., Bouiadjra B.A.B. (2012). Finite element analysis of the thermal residual stresses of SiC particle reinforced aluminum composite. Comput. Mater. Sci..

[8] Li L., Han Z., Gao M., Li S., Wang H., Kang H., Guo E., Chen Z., Wang T. (2022). Microstructures, mechanical properties, and aging behavior of hybrid-sized TiB2 particulate-reinforced 2219 aluminum matrix composites. Mater. Sci. Eng. A.

[9] Ahmadi M., Sadighi M., Hosseini-Toudeshky H. (2022). Microstructure-based deformation and fracture modeling of particulate reinforced composites with ordinary state-based peridynamic theory. Compos. Struct..

[10] Ali R., Ali F., Zahoor A., Shahid R.N., Tariq N.H., He T., Shahzad M., Asghar Z., Shah A., Mahmood A. (2021). Effect of sintering path on the microstructural and mechanical behavior of aluminum matrix composite reinforced with pre-synthesized Al/Cu core-shell particles. J. Alloys Compd..

[11] Shao J.C., Xiao B.L., Wang Q.Z., Ma Z.Y., Yang K. (2011). An enhanced FEM model for particle size dependent flow strengthening and interface damage in particle reinforced metal matrix composites. Compos. Sci. Technol..

[12] Qing H. (2014). Micromechanical study of influence of interface strength on mechanical properties of metal matrix composites under uniaxial and biaxial tensile loadings. Comput. Mater. Sci..

[13] Qing H. (2013). Automatic generation of 2D micromechanical finite element model of silicon–carbide/aluminum metal matrix composites: Effects of the boundary conditions. Mater. Des..

[14] Qing H. (2013). 2D micromechanical analysis of SiC/Al metal matrix composites under tensile, shear and combined tensile/shear loads. Mater. Des..

[15] Yan Y.W., Geng L., Li A.B. (2007). Experimental and numerical studies of the effect of particle size on the deformation behavior of the metal matrix composites. Mater. Sci. Eng. A.

[16] Zhang X., Chen T., Ma S., Qin H., Ma J. (2021). Overcoming the strength-ductility trade-off of an aluminum matrix composite by novel core-shell structured reinforcing particulates. Compos. Part B Eng..

[17] Zhang W.X., Li L.X., Wang T.J. (2007). Interphase effect on the strengthening behavior of particle-reinforced metal matrix composites. Comput. Mater. Sci..

[18] Geni M., Kikuchi M. (1998). Damage analysis of aluminum matrix composite considering non-uniform distribution of SiC particles. Trans. Jpn. Soc. Mech. Eng. Ser. A.

[19] Chawla N., Ganesh V.V., Wunsch B. (2004). Three-dimensional (3D) microstructure visualization and finite element modeling of the mechanical behavior of SiC particle reinforced aluminum composites. Scr. Mater..

[20] Sozhamannan G.G., Prabu S.B., Paskaramoorthy R. (2010). Failures analysis of particle reinforced metal matrix composites by microstructure based models. Mater. Des..

[21] Sozhamannan G.G., Prabu S.B. (2009). Influence of interface compounds on interface bonding characteristics of aluminium and silicon carbide. Mater. Charact..

